# Single molecule technique unveils the role of electrostatic interactions in ssDNA–gp32 molecular complex stability†

**DOI:** 10.1039/d3ra07746b

**Published:** 2024-02-13

**Authors:** Irina Schiopu, Isabela Dragomir, Alina Asandei

**Affiliations:** a The Institute of Interdisciplinary Research, Department of Exact Sciences and Natural Sciences, “Alexandru Ioan Cuza” University of Iaşi 700506 Iasi Romania alina.asandei@uaic.ro

## Abstract

The exploration of single-strand DNA-binding protein (SSB)–ssDNA interactions and their crucial roles in essential biological processes lagged behind other types of protein–nucleic acid interactions, such as protein–dsDNA and protein–RNA interactions. The ssDNA binding protein gene product 32 (gp32) of the T4 bacteriophage is a central integrating component of the replication complex that must continuously bind to and unbind from transiently exposed template strands during the DNA synthesis. To gain deeper insights into the electrostatic conditions influencing the stability of the ssDNA–gp32 molecular complex, like the salt concentration or some metal ions proven to specifically bind to gp32, we employed a method that performs rapid measurements of the DNA–protein stability using an α-Hemolysin (α-HL) protein nanopore. We indirectly probed the stability of a protein–nucleic acid complex by monitoring the dissociation process between the gp32 protein and the ssDNA molecular complex in single-molecular electrophysiology experiments, but also through fluorescence spectroscopy techniques. We have shown that the complex is more stable in 0.5 M KCl solution than in 2 M KCl solution and that the presence of Zn^2+^ ions further increases this stability for any salt used in the present study. This method can be applied to other nucleic acid–protein molecular complexes, as well as for an accurate determination of the drug–protein carrier stability.

## Abstract 2

A single-molecule electrophysiology assay was employed using an α-HL nanopore to test the stability of ssDNA and gp32 SSB–protein, in different salt conditions. Low salt and the presence of Zn^2+^ increases ssDNA–gp32 complex stability.

## Graphical abstract

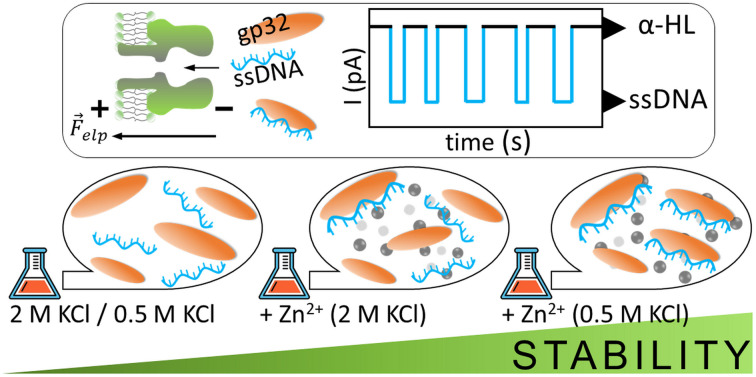

## Introduction

The most vital biochemical reactions in active cells, such as DNA replication, accurate transcription, processing, repair, specific package, and DNA rearrangement require sustainable and functional interactions between nucleic acids and specific proteins. To provide cellular genome maintenance machinery access to genomic information, DNA must be unwound to form single-stranded (ss) intermediates.^1^ Several proteins work in collaboration to successfully open up the DNA double helix (*e.g.*, enzyme helicase)^2^ and effectively create a replication fork of two single stranded regions of the DNA, which are available as templates for the synthesis of new daughter strands (a process catalyzed by DNA polymerases). Single-stranded DNA-binding proteins (SSBs) bind to the exposed regions of ssDNA to stabilize the separated strands and to prevent the DNA from adopting unfavorable conformations.^3^ Contacts between DNA and proteins involve noncovalent interactions, stabilizing the resulting molecular complex to execute essential biological functions. However, these interactions also allow for the facile disassembly of the complex, enabling both biomolecules to contribute additional functions to the cell. The noncovalent contacts between DNA and proteins have traditionally been categorized as hydrogen bonding (direct or water-mediated), ionic (salt bridges or DNA backbone interactions) and other forces, including van der Waals and hydrophobic interactions.^4,5^ Molecular complexes between SSBs and ssDNA are known for their significant thermodynamic stability, resulting from electrostatic interactions.^6–8^ These occur between the negatively charged phosphate groups in ssDNA and the positively charged amino acid residues, such as lysine (K), arginine (R), or histidine, present in SSBs' binding sites.^9^ While a robust electrostatic interaction is crucial for SSBs to stabilize ssDNA, their transient nature in DNA-related processes requires the ability to detach and reattach to ssDNA. Additionally, SSBs must reposition themselves within ssDNA complexes.^10^ The current understanding of SSB–ssDNA interactions mainly comes from several extensively studied individual SSBs,^11^ such as bacteriophage T4 gene 32 protein (gp32),^12–15^*Escherichia coli* SSB,^16–18^ replication factor A (RPA)^19–22^ and human SSB1 and SSB2.^23^ Stable complexes between proteins and nucleic acids are essential. When these complexes become excessively stable, it can prevent reforming the double-stranded DNA (dsDNA). This disruption in the formation of dsDNA is noted as a potential consequence and is associated with various diseases, including neurodegenerative disorders and cancers. Therefore, the balance in the stability of these complexes is essential for cellular health, since too stable complexes or unstable complexes can have harmful effects on important biological processes.^24^

The plethora of techniques developed for studying SSB–ssDNA interactions has made the selection of the appropriate method a challenging task. Currently, several methods are available for investigating these interactions, including: (i) electrophoretic mobility shift assays,^25^ (ii) isothermal titration calorimetry, (iii), surface plasmon resonance, (iv), chromatin immunoprecipitation,^26^ (v) fluorescence methods^27,28^ and (vi) optical tweezers and atomic force microscopy (AFM).^29^ Nevertheless, fluorescence single-molecule experiments revealed diverse conformational sub-states within protein–DNA complexes that aren't easily observable with other methods.^30–32^ Solid-state nanopores have also been employed for detecting molecular complexes of DNA with other small molecules or biomolecules. Various synthetic nanopores have been used to detect and quantify virus/antibody and protein/antibody complexation.^33–39^ Nanopore sensing is one of the latest additions to the growing arsenal of single molecule-methods.^40^ The single-molecule detection technique using nanopores enables the direct and real-time detection of a wide range of molecules, with low cost and minimal material consumption.^41^ This process typically involves electrophoretic forces, which can draw electrically charged individual DNAs or proteins to the sensing region of the nanopore, allowing efficient sampling of the molecules from a given specimen.^42^ The operating principle of this approach generally follows this sequence of events: (a) by applying a potential difference across a nanopore inserted in a lipid membrane, an ionic current is generated from K^+^ and Cl^−^ ions resulting from the dissociation of the KCl salt in the electrolytic solution, measured along the ionic channel; (b) the electric field stemming from the potential difference guides the molecule of interest toward the nanopore; (c) the transient capture of the molecule inside the nanopore involves changes in the electrical resistance of the nanopore, perceived as fluctuations in the ionic current mediated by the nanopore.^43–47^

To decipher what the factors that influence the stability of the protein–DNA complexes are, single-molecule approaches have become a powerful resource. One extensively studied model for such interactions is the Gene 32 protein (gp32), the single-stranded (ss) DNA binding protein of the bacteriophage T4, a Zn^2+^ metalloprotein.^48,49^ It transiently and cooperatively binds to exposed sequences of ssDNA during the DNA replication process, regulating interactions between other sub-assemblies of the replication complex throughout the replication cycle.^12^ gp32 is thought to be able to quickly cover those transiently single stranded regions that arise near the advancing T4 DNA replication forks and in so doing stabilizes a particular ssDNA conformation that is most appropriate to serve as a substrate for other catalytic proteins.^50^ Biochemical insights obtained from studies of gp32 continue to serve as an important basis for understanding the function of these proteins in bacteria and other higher organisms.^51^ The gp32 protein has an N-terminal domain, a C-terminal domain, and a core domain. The N-terminal domain is necessary for the cooperative binding of the gp32 protein through its interactions with the core domain of an adjacent gp32 protein. To bind to ssDNA, the C-terminal domain of the gp32 protein must undergo a conformational change that exposes the positively charged region of its core domain, which in turn, interacts with the negatively charged ssDNA backbone. The binding site size of the gp32 protein is seven nucleotide residues long.^52^ The complexes formed between proteins and ssDNA are more difficult to predict, considering the following significant details: (i) the lack of a definite structure of the ssDNA molecules and their great flexibility and, (ii) the large heterogeneity of the SSB–ssDNA interface. The disordered nature of ssDNA enables it to interact with proteins mostly through the phosphate groups, which may attract charged amino acid side chains; or the bases, which may interact with the aromatic amino acid side chains, with a consequent increase in interface heterogeneity.^6^ Moreover, gp32 protein core can adopt multiple conformations, depending on the different numbers of nucleotides engaged.^29^*In vivo*, the stability of DNA will be affected by a number of factors beyond the presence of SSB proteins. Variations in buffer content and temperature may affect the DNA itself as well as the binding behavior of the proteins.^53^

Herein we employed an indirect method using a protein nanopore to decipher what the factors which may perturb the ssDNA–gp32 complex are. We demonstrated that varying salt concentration affects the stability of the ssDNA–gp32 complex, and furthermore, the presence of Zn^2+^ ions enhance complex stability compared to cases without the metal ion. Using single molecule recordings, we can simplify the system we are studying. Understanding some of the factors that affect the ssDNA–protein stability will open the door for the development of new medicinal and biological applications, including rational drug design and the control of gene expression. Also, studies can be extended to solution conditions not available to bulk studies, allowing us to gain new insights into specific DNA–ligand interactions in biological systems.

## Materials and methods

### Reagents and chemicals

ssDNA was procured from Sigma-Aldrich, Germany, with a 16-nt long primary sequence: 5′-ACG GAA GGA GTG CCA A-3′ (*M*_w_ = 4968 g mol^−1^). The other reagents, including the T4 Gene 32 Protein (gp32) from *Escherichia coli* (infected with phage T4amN134/amBL292/amE218) solution (in storage buffer 20 mM Tris–HCl, 100 mM NaCl, 1 mM EDTA, 0.5 mM DTT, 50% glycerol [v/v], pH approximately 8.0), α-hemolysin (α-HL) monomeric protein, potassium chloride (KCl), zinc chloride (ZnCl_2_) sodium chloride (NaCl), EDTA, ultra-pure water (DNAase and RNAase free), Tris and HEPES buffers, potassium hydroxide (KOH), hydrochloride acid (HCl), *n*-pentane and the hexadecane – were purchased from Sigma-Aldrich, Darmstadt, Germany. The 1,2-diphytanoyl-*sn-glycero*-phosphocholine lipid (DPhPC) used for forming the artificial lipide membrane in the single-molecule experiments was purchased from Avanti Polar Lipids, Alabaster, AL, USA.

### Sample preparation

The ssDNA sample in dried form was dissolved in 1 M NaCl solution in ultra-pure water buffered with TE (10 mM Tris, 1 mM EDTA) at pH = 8.2 and vigorously stirred using a Stuart BioCote vortex mixer (Sigma-Aldrich, Germany) for 3 minutes at 1.400 rpm, in continuous mode, to obtain a stock solution of 100 μM. The sample was then heated up to 95 °C for 20 min and slowly cooled down to 23 °C in order to improve the rehydration. Aliquots of solution were transferred into new vials and were stored at −20 °C until further use. Before being used in electrophysiology experiments or fluorescence experiments, aliquots from the ssDNA solution were annealed by rapidly heating them to 95 °C using an IKA Digital Block Heater (Cole-Parmer, USA) and slowly cooled down to 23 °C. The T4 Gene 32 Protein (gp32) (stock concentration 164 μM) was used as purchased. The ssDNA and gp32 protein mixture or ssDNA and gp32 and ZnCl_2_ mixture was incubated at a molar ratio of 1 : 2, and 1 : 2 : 20 for 3 h at 23 °C respectively.

### Nanopore electrophysiology

The single-molecule nanopore recordings followed previously described protocols.^46^ Bilayer lipid membranes (BLMs) were obtained by employing the Montal–Muller technique.^54,55^ Briefly, the 1,2-diphytanoyl-*sn-glycero*-phosphocholine dissolved in *n*-pentane formed stable solventless bilayers across a ∼120 μm in diameter orifice punctured on a 25 μm-thick Teflon film (Goodfellow, Malvern, MA) that was pretreated with 1 : 10 hexadecane/pentane. The recording chamber, consisting of two compartments denoted by *cis* (grounded) and *trans*, was filled with different electrolyte solutions buffered at pH 7 used according to the experimental protocol (*i.e.*, 0.5 M KCl and 2 M KCl). To improve the signal-to-noise ratio of single molecule nanopore-based measurement we must use higher-salt experimental conditions (≥0.5 M). The addition of ∼0.5 to 2 μL α-HL from a monomeric stock solution made in 0.5 M KCl to the grounded compartment (*cis*) under ∼10 min continuous stirring led to the insertion of a single heptameric α-HL nanopore into the previously formed stable lipid membrane. The molecules ssDNA, gp32 protein, Zn^2+^, ssDNA–gp32 incubated complex (molar ratio 1 : 2) and ssDNA–Zn^2+^–gp32 incubated complex (molar ratio 1 : 20 : 2) were added at a 100 nM, 2 μM, and 200 nM respectively on the *cis* side of the nanopore. The ion current fluctuations across the α-HL nanopore reflecting unimolecular interactions between the ssDNA–nanopore interactions or the gp32–nanopore interactions or the ssDNA–gp32 complex–nanopore interactions or the ssDNA–Zn^2+^–gp32 complex–nanopore interactions were recorded at positive potential differences Δ*V* = +150 mV across the membrane, in the voltage-clamp mode with an Axopatch 200B (Molecular Devices, Sunnyvale, CA) amplifier. The amplified signals were low-pass filtered at 10 kHz and digitized with a NI PCI 6221, 16-bit acquisition board (National Instruments, USA, Austin, TX) at a sampling frequency of 50 kHz, with a virtual instrument developed within the LabVIEW 8.20 (National Instruments, USA, Austin, TX). All measurements were carried out at a room temperature of ∼23 °C. Data analysis was undertaken with the Origin 6 (OriginLab, Northampton, MA) and pClamp 6.03 (Axon Instruments, Union City, CA) software as previously described.^56^

### Fluorescence spectroscopy

To complementarily probe the salt influence on ssDNA–gp32 complex dissociation, we performed fluorescence spectroscopy experiments using a FluoroMax-4 spectrofluorometer (Horiba, Jobin Yvon, USA). In these experiments, 1 mL of reference solution (0.5 M KCl or 2 M KCl buffered with 10 mM HEPES at pH ∼ 7) was pipetted in a quartz cuvette with a 10 mm path length. After the addition of 200 nM gp32 protein into the cuvette, the emission fluorescence spectrum was recorded at an excitation wavelength of 290 nm. Furthermore, the variation over time of the maximum fluorescence intensity at 340 nm was monitored for the pre-incubated ssDNA–gp32 mixture at a molar ratio of 1 : 2, for a total of 45 minutes time interval, every 5 minutes. The recorded spectra were further analyzed and presented with Origin 6 (OriginLab, Northampton, MA) software.

## Results and discussion

### Design of the study

Our objective was to investigate the real-time interaction between gp32 and DNA at the single molecule level, utilizing an α-HL nanopore. Following the insertion of the nanopore into a lipid bilayer, we added the gp32 protein in the *cis* compartment of the BLM cell. A positive transmembrane electrical potential was applied in order to electrophoretically pull the gp32 at the nanopore's mouth enabling thus the real-time monitoring of the α-HL–gp32 protein–protein interactions.

**Fig. 1:**
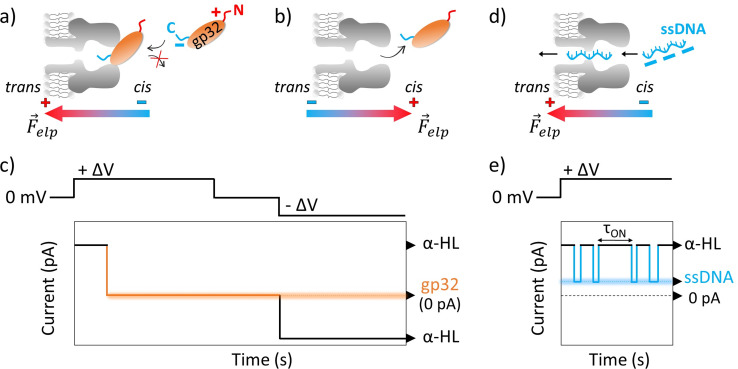
Schemas depicting the irreversible α-HL–gp32 protein interactions *versus* the reversible α-HL–ssDNA interactions. Shortly after applying a transmembrane voltage, the overall negatively charged gp32 protein (*Q* = −10|*e*^−^| at pH 7 (ref. 57)) is electrophoretically driven towards the nanopore's *cis*-opening with its negatively C-terminus domain, irreversibly and totally (*I* = 0 pA in an idealized scenario) blocking the nanopore's mouth (panel a and c), while the core's protein and its positively charged N-terminus domain remain outside the nanopore. Once the applied voltage polarity is reversed, the gp32 protein will be released from the nanopore (panel b and c) and the current value will return to its initial value, corresponding to the free-nanopore (α-HL). In contrast, the negatively charged ssDNA molecules are captured and driven through the nanopore by the electrophoretic force (*F⃑*_elp_) (panel d), which gives rise to transient reversible current blockage events (panel e) specific to a ssDNA molecule, characterized by *τ*_ON_ (panel e) representing the time interval between subsequent events.

Upon increasing the gp32 protein concentration we observed an almost total and irreversible blockage of the nanopore by the protein (Fig. 1a), unless the polarity of the applied transmembrane potential was reversed (Fig. 1b and c).

The probability of the nanopore obstruction by the gp32 protein increased considerably with the presence of a higher gp32 protein concentration in the *cis* compartment (data not shown). Thus, an optimal gp32 concentration of 200 nM was established to ensure that the nanopore remains free for a suitable time interval needed for the ongoing gp32–DNA experiments. Also, the gp32 protein remains in the monomer state. The irreversible blockage of the nanopore by the protein at positive potentials can be attributed to: (i) geometrical limitation; (ii) the electric charge distribution and (iii) the secondary structure of the gp32 protein. The physical shape of the gp32 protein is one of a prolate ellipsoid with an axial ratio of 4 : 1 (12 nm length and 3 nm width),^12,58^ making the gp32 protein too large to enter in the nanopore vestibule with an opening of 2.6 nm, which narrows slightly to 2.4 nm and then widens into the interior vestibule at 3.6 nm.^59^ This geometric mismatch holds the gp32 protein atop the α-HL nanopore. Moreover, based on the amino acid sequence, the gp32 protein^49^ has a negative electric charge of −10 at pH 7,^57^ which is in agreement with an experimentally determined isoelectric point of 5.0. The electric charge distribution of the gp32 is quite asymmetric: while the NH-terminal domain has a net positive charge (+10|*e*^−^|), the COOH-terminal domain has a net negative charge (−20|*e*^−^|).^57^ Furthermore, the molecule can be divided into three regions: the amino terminal (residues 1–35) and the carboxyl terminal (residues 187–301) sequences which appear to have mainly an α-helical secondary structure, and the middle (the core) domain which has primarily a β-sheet structure.^60^

### Single-molecule investigation of salt concentration effect on the ssDNA–gp32 molecular complex stability

Gene 32 protein in solution in the absence of DNA binding targets exists primarily as protein monomers. The ‘core’ or central portion of the gp32 monomer is the ssDNA-binding domain and comprises 235 amino acid residues. This core domain contains an oligonucleotide–oligosaccharide binding fold – a motif generally found in ssDNA binding proteins—and confers ssDNA binding specificity and polarity onto the gp32 molecule. The N-terminal (20 amino acid residues) domain of the gp32 protein is required for the cooperative binding of gp32 monomers to long ssDNA lattices and the C-terminal domain (46 residues) being essential for the regulatory interactions of gp32 with other proteins of the T4-coded DNA replication, recombination and repair complexes.^13,60^ Thus, after establishing how the α-HL–gp32 protein–protein interplay takes place, we performed experiments in which we followed the α-HL–ssDNA interactions. The ssDNA molecules were added in the *cis* side of the nanopore, and the resulting current fluctuations produced following the nanopore-ssDNA interactions at a positive potential difference (+Δ*V*) were recorded (Fig. 1d and e). The parameter measured in the experiments was the inter-event (*τ*_ON_) measured in seconds, representing the time interval between subsequent α-HL–ssDNA interactions, which is an indicator for these interactions under any given conditions. Experiments were conducted in solutions with either 2 M KCl solution or 0.5 M KCl solution, buffered with 10 mM HEPES at pH = 7. The constant rates (rate_ON_ = 1/*τ*_ON_) of α-HL–ssDNA interactions obtained were: 2.688 ± 0.180 s^−1^ in 2 M KCl solution and 1.219 ± 0.123 s^−1^ in 0.5 M KCl solution. A subsequent step involved the addition of the gp32 protein with the ssDNA at a molar ratio of 1 : 2 ssDNA : gp32. By adding the gp32 protein in the same compartment where the ssDNA is already present, we sought to investigate potential correlations between the protein's presence and the observed current fluctuation patterns. Upon the addition of gp32 molecules to the system, we expected two main changes to happen: (i) a reduction in the ssDNA interaction events caused by a decrease in the DNA concentration due to its binding to the protein, and (ii) the emergence of other types of events such as bumping, due to the excessively large size of the ssDNA–gp32 complexes formed.^61^

**Fig. 2:**
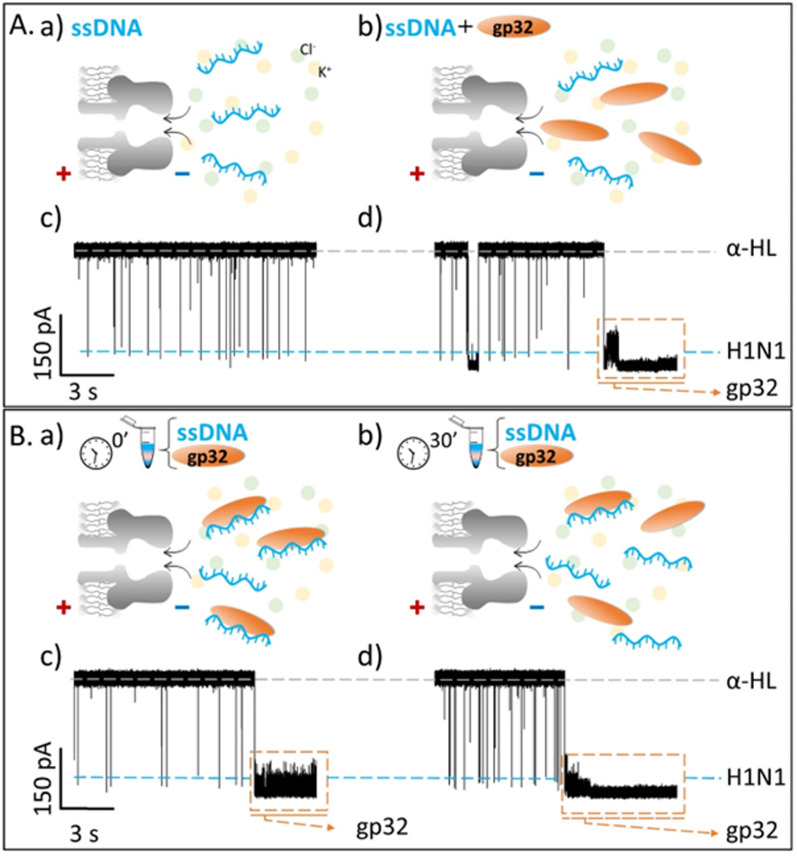
Single-molecule electrophysiology recordings in a 2 M KCl solution. (A) The recordings showing reversible current blockage events of the negatively charged 100 nM ssDNA, added in *cis*-side of the nanopore before (panels a, c) and after (panels b, d) the *cis*-side addition of 200 nM gp32 protein, non-incubated. (B) The current fluctuations recorded for the 3 h pre-incubated ssDNA : gp32 at 1 : 2 moral ratio added in the *cis*-side of the nanopore at the initial time (panels a, c) and after 30 minutes of recording (panels b, d). All shown original traces were recorded at +150 mV transmembrane potential, in a pH 7, 10 mM HEPES buffered solution. The dashed gray lines represent the open state of the α-HL nanopore; the blue-glow lines represent the blockage level given by the ssDNA, and the orange-dashed frame represents the irreversible association of gp32 protein with the nanopore.

**Fig. 3:**
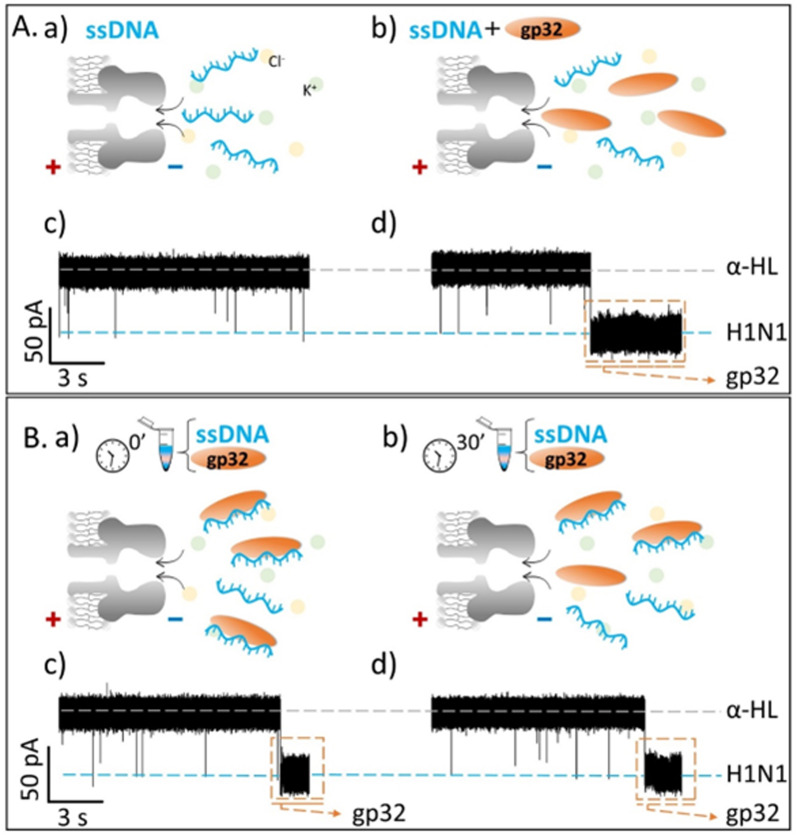
Single-molecule electrophysiology recordings in a 0.5 M KCl solution. (A) The recordings showing reversible current blockage events of the negatively charged 100 nM ssDNA added in *cis*-side of the nanopore before (panels a, c) and after (panels b, d) the *cis*-side addition of 200 nM gp32 protein, non-incubated. (B) The current fluctuations recorded for the 3 h pre-incubated ssDNA : gp32 at 1 : 2 moral ratio added in the *cis*-side of the nanopore at the initial time (panels a, c) and after 30 minutes of recording (panels b, d). All shown original traces were recorded at +150 mV transmembrane potential, in a pH 7, 10 mM HEPES buffered solution. The dashed gray lines represent the open state of the α-HL nanopore; the blue-glow lines represent the blockage level given by the ssDNA, and the orange-dashed frame represents the irreversible association of gp32 protein with the nanopore.

Contrary to what we expected, we observed that the recorded ionic current fluctuations were specific to either the ssDNA molecules or the gp32 proteins (Fig. 2Ab, d and Fig. 3b, d). Therefore, a very important question emerged: *do the ssDNA and the gp32 form molecular complexes under the established experimental conditions? If so, why were the complexes not observable by the nanopore technique in a direct manner?* Thus, by changing the paradigm, the ssDNA and gp32 molecules, in a molar ratio of 1 : 2, were incubated for 3 hours, at room temperature, and further added in the *cis*-side of the nanopore. The single-molecule recordings of the incubated mixture were monitored, and, initially, a low ssDNA–nanopore interaction events frequency was observed. The blockage events were mainly specific to the ssDNA finger-print recorded in the control experiments (Fig. 2Ac and Fig. 3Ac) but with a considerably lower frequency (Fig. 2Bc and Fig. 3Bc). After 30 minutes, without extra-intervening upon the system by stirring or other means, a significant increase in the blockage events frequency was observed. All the recordings were performed at a +150 mV transmembrane potential.

By comparing the two experiments performed in two different salt solutions, 2 M KCl (Fig. 2Bb and d) and 0.5 M KCl (Fig. 3Bb and d), we noticed that after 30 minutes the events frequency is higher in the former case. In both cases, only the ssDNA translocates through the nanopore, which is wide enough to accommodate the ssDNA but not the ssDNA–gp32 molecular complex.

The fully reproducible experiments gave the same results each time: the ssDNA blockage events frequency increased over time (Fig SI_2†), notably at high salt than at low salt concentration. The relative growth rate for a 0.5 M KCl solution was 0.275 ± 0.075, and 2.662 ± 0.403 for the 2 M KCl solution, almost 9-fold higher than in low salt conditions. Also, the relative ionic current blockages (Δ*I*/*I*_O_) for α-HL–ssDNA and α-HL–gp32 interaction in both low (0.5 M KCl) and high (2 M KCl) salt conditions were calculated (Table 1), based on the difference (Δ*I* = *I*_open_ – *I*_blockage_) between the recorded free-nanopore ionic current (*I*_open_) and the blockage ionic current given by either ssDNA or gp32–protein (*I*_blockage_) relative to the free-nanopore current (Fig SI_3†). In both salt conditions, the differences can be clearly observed not only in the recorded current fingerprint of each molecule (i.e., ssDNA or gp32), but also in the relative ionic current blockage values obtained; the gp32 protein blockage being always higher than the ssDNA.

**Table 1:** The relative ionic current blockage (Δ*I*/*I*_O_) of the α-HL nanopore by ssDNA and gp32 in 0.5 M and 2 M KCl salt concentration

Salt conditions	Relative ionic current blockage^a^ (Δ*I*/*I*_O_) for:	
0.5 M KCl	ssDNA	0.79 ± 0.01
	gp32	0.83 ± 0.01
2 M KCl	ssDNA	0.86 ± 0.01
	gp32	0.91 ± 0.02

a The relative ionic current blockage calculated by formula: Δ*I*/*I*_O_ = (*I*_open_ – *I*_blockage_)/*I*_open_.

The single-molecule electrophysiology experiments, using the α-HL biological nanopore as a nanosensor, showed that: (i) ssDNA–gp32 molecular complexes are best formed by incubation; (ii) the molecular complexes dissociate over time and (iii) the stability of the ssDNA–gp32 complex is highly influenced by the salt concentration of the used physiological solution.

### The confirmation of the salt influence on the stability of the ssDNA–gp32 complex through fluorescence spectroscopy measurements

The fluorescence-quenching physicochemical process is one of the most extensively used techniques to measure the affinity of the gp32 protein for single-stranded nucleic acids.^57^ Herein we performed spectrofluorometric measurements to verify and confirm the data obtained from the single-molecule electrophysiology experiments according to which the salt concentration of the solution has an extensive influence over the stability of the ssDNA–gp32 protein complexes formed by incubation.

**Fig. 4:**
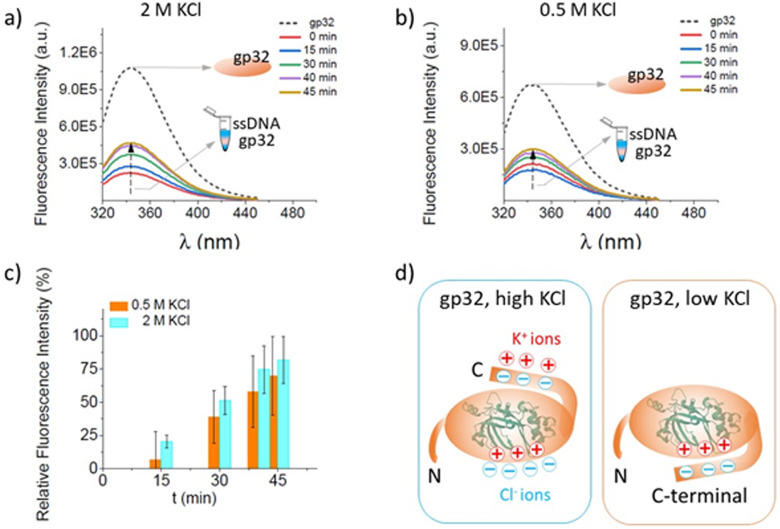
Fluorescence spectra changes recorded in time, in a 2 M KCl (panel a) and 0.5 M KCl (panel b) solution, for gp32 protein without the ssdna (black, dashed-line) and pre-incubated with the ssDNA (colored, continuous-line) at a molar ratio of 1 : 2 ssDNA : gp32. The relative increase in the fluorescence intensity with time (panel c) for 2 M KCl (cyan columns) and 0.5 M KCl (orange columns) salt condition reflecting the ssDNA–gp32 molecular complex dissociation. Schematic representation of the predominant possible conformations for the gp32 protein in high and low salt conditions (panel d). In high salt, the C-terminal of gp32 is unbound from the core, and three potassium cations are condensed onto it, while four chloride ions are condensed onto the cationic DNA binding site. In low salt, the C-terminal of gp32 is bound to the cationic DNA binding site (adapted from ref. 62).

The increase in the maximum fluorescence emission spectrum of the Trp amino acids, present in the primary structure of the gp32 protein, has been monitored and recorded (Fig. 4a and b). The increase of the fluorescence intensity was associated with conformational changes of the protein upon its dissociation from the ssDNA molecule. The gp32 protein (Fig. 4a and b, dashed-line) fluorescence emission spectrum was recorded first, followed by the recording of the emission fluorescence spectra of the incubated ssDNA–gp32 molecules (in a molar ratio of 1 : 2) with a 5-minute iteration time. An increase in fluorescence intensity over time was observed, which tends towards the maximum fluorescence intensity recorded for the gp32 protein alone. This suggested a complete dissociation of the ssDNA–gp32 complex, albeit only after a lengthy recording time (*i.e.* ∼hours, data not shown). By correlating the results obtained by these two applied methods, electrophysiology and spectrofluorometric, we can conclude that the stability of the incubated formed ssDNA–gp32 molecular complex depends highly on the salt concentration of the solution in which they are immersed.

The relative fluorescence intensity growth depicted in Fig. 4 panel c was calculated for both 0.5 M KCl and 2 M KCl solution, using the formula below: 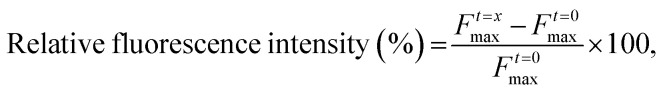
where *F*^*t*=*x*^_max_ represents the maximum fluorescence intensity at a certain time in the recording (*t* = *x* min), and *F*^*t*=0^_max_ represents the maximum fluorescence intensity at the initial time (*t* = 0 min).

One common feature of the protein-nucleic acid interactions is their strong dependence on the salt levels when studied *in vitro*.^63^ In this context, as the salt concentration increases, the observed stability significantly decreases. This phenomenon is consistent in any interaction between a positively charged molecule and a linear nucleic acid, and it arises from the polyelectrolyte nature of the linear nucleic acid. Research has shown that the highly negative charge along the backbone of a linear nucleic acid leads to the accumulation of counterions, such as potassium ions (K^+^), located in close proximity to the nucleic acid, which partially neutralize the densely packed phosphate groups in the DNA's backbone.^64^ As the monovalent salt concentration is increased, the electrostatic shielding is strengthened and the ion condensation-dependent mixing entropy effects are reduced; thus, a new class of ssDNA conformations – which are stabilized by high salt and which disfavors gp32 binding to ssDNA – appear.^3^ The conformational flexibility of either ssDNA or gp32 protein can affect the predicted structures. In the case of ssDNA, its flexibility is linked to electrostatic forces and can, therefore, be modulated by salt concentration.^13,65^

However, the salt dependence of ligand binding to nucleic acids can be described in the context of counterion condensation theory, which was first derived by Oosawa^66,67^ and Manning^68^ for the hypothetical case of an infinite line charge. They have shown that, counterions can condense onto an electrically charged macromolecule (*e.g.*, DNA, proteins), changing its surrounding charge density. The counterions found in the vicinity of a DNA molecule are effectively bound to it. Therefore, for a charged ligand to bind to the DNA, these counterions must be removed, resulting in a free energy change due to the release of the counterions back into the solution. The physical basis of such a phenomenon is in fact long established through the Record–Lohman equation, expressing the ionic strength dependence of the binding constant characterizing DNA interactions with oligopeptides and proteins.^69,70^ The binding site size (*n*) of a gp32 monomer for a ssDNA molecule has a 7 nucleotide residues length^71^ and it binds in cooperative clusters to long ssDNA lattices at approximately physiological salt concentrations. In the absence of cooperativity, the direct binding of individual gp32 molecules to ssDNA lattices is primarily stabilized by electrostatic interactions between positively charged amino acid side chains within the protein binding site and the negatively charged phosphates of the ssDNA backbone. This direct affinity of isolated gp32 molecules for ssDNA is strongly dependent on salt concentration, reflecting the significant free energy contributions of the displacement of condensed counterions from the phosphates by the multivalent positively charged gp32 binding site.^71,72^

### ssDNA–gp32 molecular complex stability enhancement in the presence of Zn^2+^ ions

**Fig. 5:**
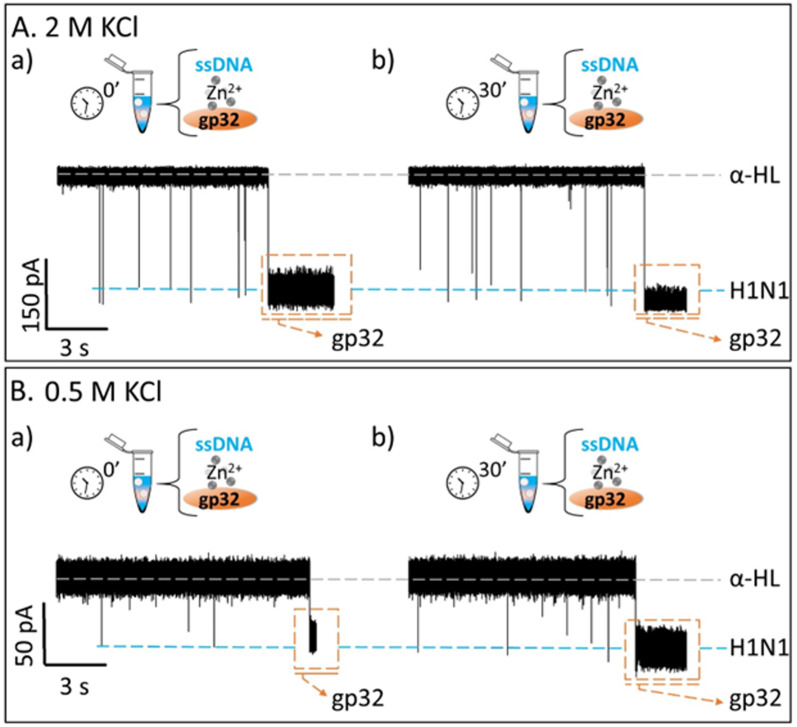
Single-molecule measurements of the current fluctuations recorded for the pre-incubated ssDNA–Zn^2+^–gp32 molecular complex in different salt concentration of the solution. (A) Experiments performed in a 2 M KCl solution, at the initial time (panel a) of the molecular complex addition, and after 30 minutes of recording time (panel b). (B) Experiments performed in a 0.5 M KCl solution, at the initial time (panel a) and after 30 minutes of recording time (panel b). The 3 h pre-incubated ssDNA–Zn^2+^–gp32 molecular complex at a molar ratio of 1 : 20 : 2 was added in the *cis*-side of the nanopore. All shown original traces were recorded at +150 mV transmembrane potential, in a pH 7, 10 mM HEPES buffered solution. The dashed gray lines represent the open state of the α-HL nanopore; the blue-glow lines represent the blockage level given by the ssDNA, and the orange-dashed frame represents the irreversible association of gp32 protein with the nanopore.

T4 gp32 protein is a Zn^2+^ metalloprotein and the removal of the Zn^2+^ metal significantly affects the structure and the DNA binding capacity of the molecule.^49^ Taking into account this property of the gp32 protein, we followed the same protocol described as above, sequentially adding ssDNA, then Zn^2+^, and finally gp32, while monitoring the nanopore blocking events. As depicted in Fig. SI_4,† there were no changes in the frequency of blocking events, indicating that no ssDNA–Zn^2+^–gp32 complexes were formed under the given conditions. The next step involved the incubation of ssDNA with Zn^2+^ and gp32 for 3 hours, followed by the addition of the incubated mixture to the *cis* compartment of the BLM cell and the recording of the resulting current fluctuations. Under these conditions, we observed that the rate of ssDNA association to the α-HL nanopore increases over time, albeit not as significantly as in the absence of Zn^2+^ ions (Fig. 5A and B). Considering the values obtained for the association rates, we can conclude that the Zn^2+^ ions confer higher stability for the ssDNA–gp32 complex in both salt conditions (*i.e.* 2 M KCl and 0.5 M KCl) (Table 2).

**Table 2:** The relative growth for the association rate between the α-HL nanopore and pre-incubated ssDNA–gp32, with and without Zn^2+^, in 0.5 M KCl and 2 M KCl solutions

Salt conditions	Relative frequency growth^a^ for:		Stability^b^
0.5 M KCl	ssDNA : gp32 (1 : 2)	0.275 ± 0.075	Good
	ssDNA : gp32:Zn^2+^ (1 : 2 : 20)	0.088 ± 0.403	Best
2 M KCl	ssDNA : gp32 (1 : 2)	2.662 ± 0.403	Worst
	ssDNA : gp32 : Zn^2+^ (1 : 2:20)	0.226 ± 0.076	Increased

a The relative growth for the association rate calculated by formula: (rate_ON_ (*t* = 30′) − rate_ON_ (*t* = 0))/rate_ON_ (*t* = 0) (see text).

b Qualitative assessment.

The relative growth for the ssDNA association rate with the α-HL nanopore as an indirect measurement for the stability of the pre-incubated ssDNA–gp32 and, ssDNA–Zn^2+^–gp32 molecular complex, respectively, in 0.5 M KCl and 2 M KCl solutions was calculated using the formula: 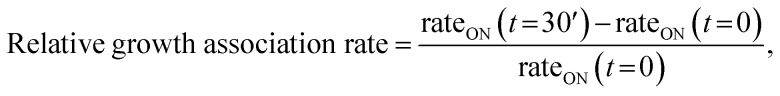
where the rate_ON_ (*t* = 30′) and rate_ON_ (*t* = 0) are the rates of association of ssDNA at the initial time (*t* = 0) of addition and after 30 minutes of recording. All the rates were calculated from the inter-events average times (*τ*_ON_) from different experiments, according to the formula: rate_ON_ = 1/*τ*_ON_. The best stability was observed as being at low salt and in the presence of zinc ions. Also, a very significant increase in stability given by the presence of zinc ions was recorded for the ssDNA–gp32 complex, at a high salted solution.

In the case of gp32, Zn^2+^ contributes to the maintenance of a suitable conformation of the DNA binding domain for the recognition of the ssDNA with high affinity in a stoichiometric fashion, establishing thus a basic structural role for Zn^2+^ in this protein, mainly due to the presence of some amino acids (i.e., Tyr and Cys) in the protein's core^49,50^ and a zinc finger consisting of His64, Cys77, Cys87 and Cys90 within the C-terminal tail that specifically interacts with Zn^2+^ ions.^15^ The removal of the metal, while not abolishing the DNA binding, does appear to weaken the interaction considerably,^73,74^ thus confirming our data.

## Conclusions

We reveal, for the first time to our knowledge, at the single-molecule level using an α-HL protein nanopore, the importance of two main factors which affect the stability of a molecular complex such as the gp32 protein–ssDNA, namely: (i) the salt concentration of the solution in which the complex can be found and (ii) the presence or absence of Zn^2+^ ions. We demonstrated the feasibility of a nanopore technique by indirectly monitoring the stability of such a molecular complex, in different conditions, making this technique applicable to other types of molecules as well. However, several issues must be addressed for this technique to be generally applicable to any protein–DNA system. Since measurements at physiological salt conditions do not provide a high enough signal-to-noise ratio, the technique is currently limited to complexes that are resistant to high-salt (>0.5 M) conditions. With further improvements and refinements, it should be possible to develop a nanopore technique for identifying DNA-binding proteins that is complementary to approaches such as chromatin immunoprecipitation (ChIP).^75^ Our results open new avenues for other paradigms that can be successfully applied using a protein nanosensor approach, including the competition between a small molecule (*e.g.*, peptides, drugs) and ssDNA for SSB proteins and enable new possibilities to investigate and control the single-molecule association of even more complicated chemistries inside nano-volumes. Answers to these questions could facilitate the rapid development of new antimicrobial drugs or personalized oncology treatments.

## Author contributions

A. A. and I. S. conceptualized the study, A. A. and I. S. acquired the funding, A. A. carried out the experiments, A. A., I. S. and I. D. oversaw the methodology and formal analysis, A. A., I. S. and I. D. oversaw the draft of the manuscript, A. A. and I. S. wrote the manuscript, A. A., I. S. and I. D. edited the manuscript, A. A. and I. S. oversaw projects administration. All authors have given approval to the final version of the manuscript.

## Conflicts of interest

There are no conflicts to declare.

## Supplementary Material

RA-014-D3RA07746B-s001
