# Supplementary material for: Single molecule technique unveils the role of electrostatic interactions in ssDNA–gp32 molecular complex stability
Source: RSC Adv. 2024 Feb 13;14(8):5449–60. doi: 10.1039/d3ra07746b (PMC10862658; doi:10.1039/d3ra07746b)
Supplement: RA-014-D3RA07746B-s001 [file RA-014-D3RA07746B-s001.pdf]

Electronic Supplementary Material (ESI) for RSC Advances.
This journal is © The Royal Society of Chemistry 2024

Single molecule technique unveils the role of electrostatic interactions in ssDNA-gp32 molecular complex stability

Irina Schiopu[#], Isabela Dragomir[#], Alina Asandei[#*]

[#]The Institute of Interdisciplinary Research, Department of Exact Sciences and Natural Sciences, „Alexandru Ioan Cuza" University of Iași, 700506 Iasi, Romania

*Corresponding author: alina.asandei@uaic.ro (A.A.)

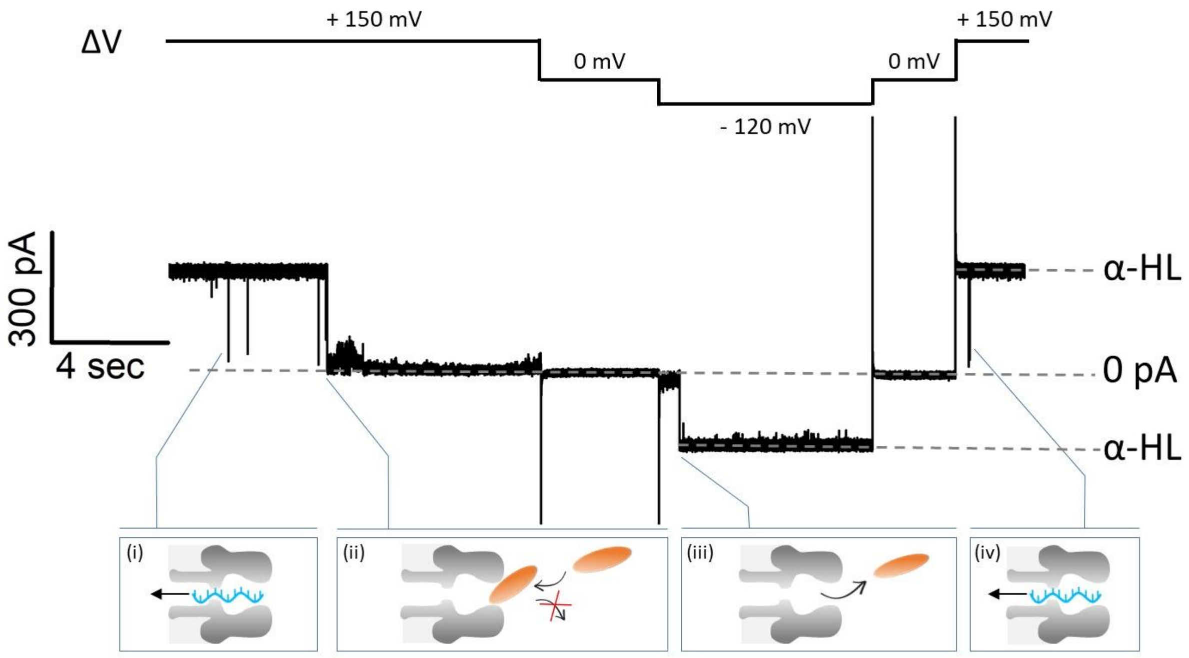

Fig_SI_1. Representative trace depicting the irreversible blockage of the α-HL nanopore by the gp32 protein. Initially, the electrophoretic force promotes the capture of the ssDNA molecules by the nanopore (panel i), until the gp32 protein irreversible blocks the nanopore's opening (panel ii). Unless the polarity of the applied electric field is reversed the overall negatively charged gp32 protein will block the nanopore (panel iii) hindering the ssDNA from entering the nanopore. Applying a negative transmembrane potential will unblock the nanopore, removing the gp32 protein, and enabling the recapture of the ssDNA molecules (panel iv).

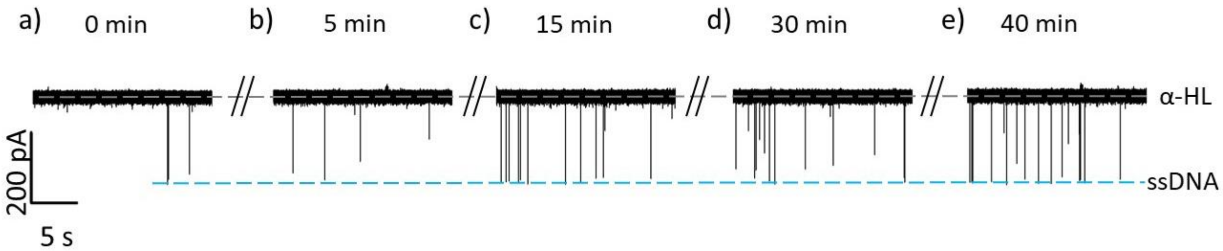

Fig_SI_2. ssDNA-gp32 molecular complex dissociation in time. Representative traces depicting the growth of ssDNA-nanopore frequency association events after the addition of the ssDNA-gp32 complex at the initial time (t = 0), and after 5, 15, 30 and, respectively, 40 minutes recording time.

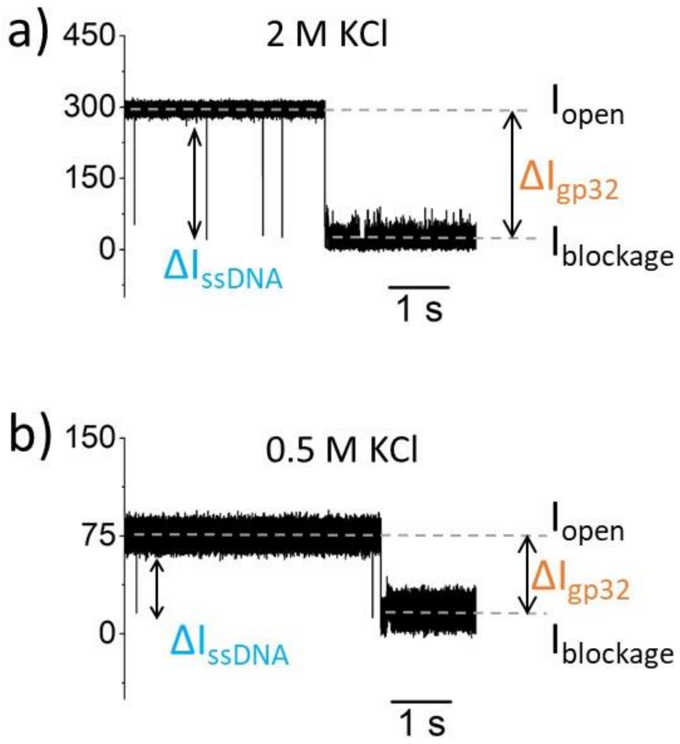

Fig_SI_3. Original traces depicting the relative ionic current blockages for α-HL-ssDNA and α-HL-gp32 interactions, in a 2 M KCl (panel a) and a 0.5 M KCl (panel b) salt solution. The difference between the free-nanopore ionic current (I_{open}) and the blockage ionic current ($I_{blockage}$) given by either ssDNA (blue ΔI_{ssDNA}) or gp32-protein (orange ΔI_{gp32}) was determined relatively to the free-nanopore current: $\Delta I / I_0 = (I_{open} - I_{blockage})/I_{open}$.

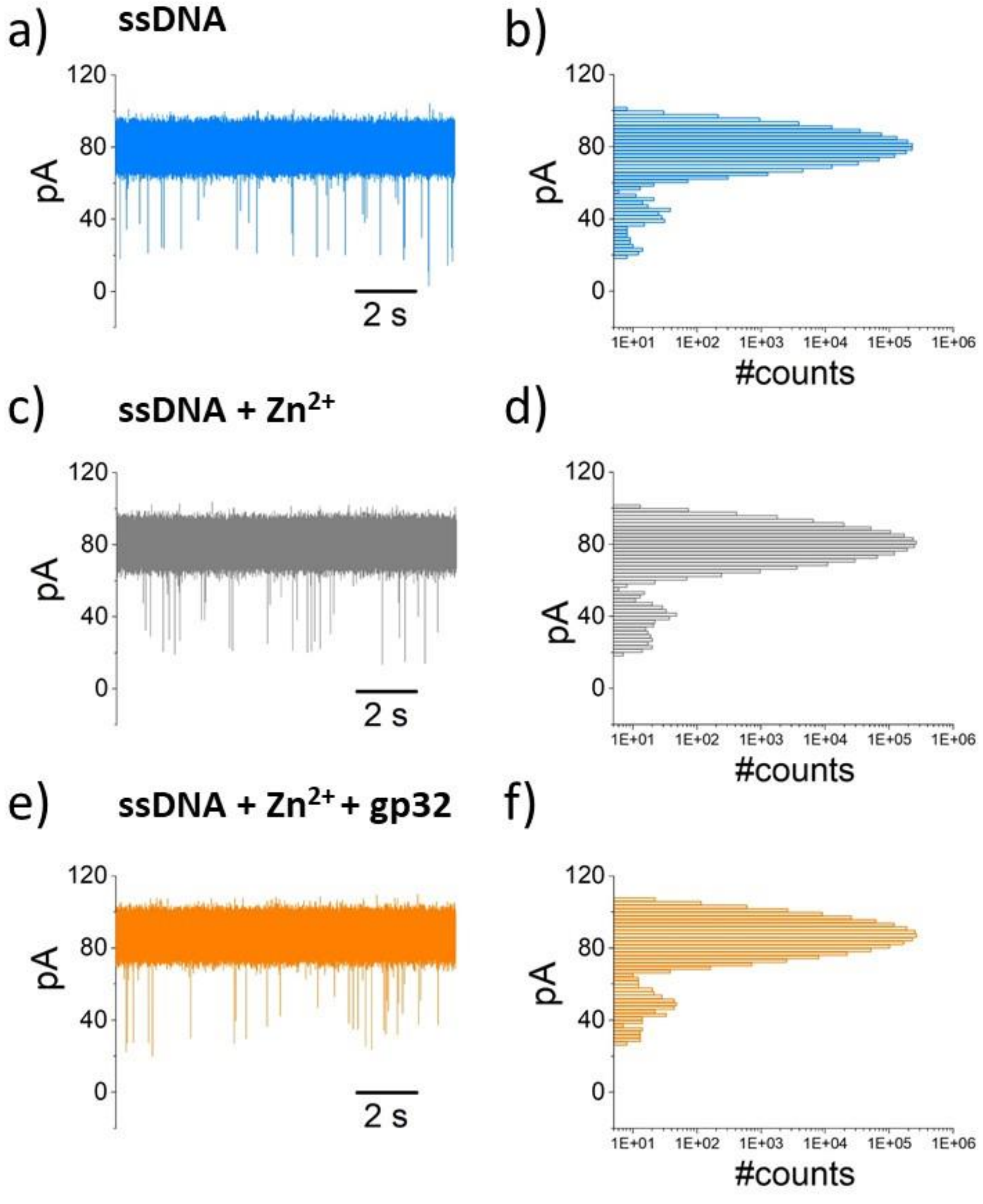

Fig_SI_4. Single-molecule control recordings in a 0.5 M KCl salt solution and their correspondent histograms for the non-incubated molecules: 0.1 µM ssDNA added in cis side of the nanopore (blue, panels a and b); 2 µM Zn^{2+} subsequently added in the cis side over the ssdna (gray, panel c and d) and 0.2 µM gp32 protein added over the ssDNA and metal ions (orange, panels e and f). All the traces depicted herein were recorded at + 150 mV.

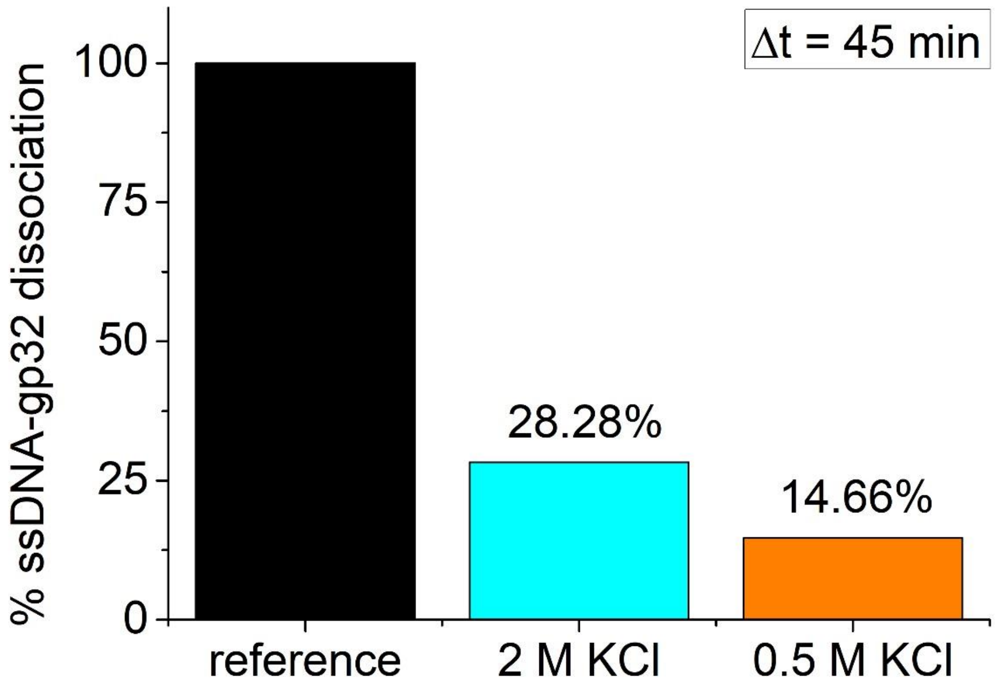

Fig_SI_5. Percentage of dissociated ssDNA-gp32 molecular complex after 45 minutes from the first recorded fluorescence emission spectrum of the pre-incubated molecular complex. We observed a higher percentage of ssDNA-gp32 complex dissociation in a 2 M KCl (cyan, 28.28%) than in 0.5 M KCl (orange, 14.66%) solution. The black column is used as a visual reference representing 100% dissociation of the molecular complex.

The percentage was calculated using the following equation:

$$\%\ ssDNA - gp32\ dissociation = \frac{F_x - F_0}{F_{max} - F_0} \cdot 100$$

where,

F_x – fluorescence intensity at a x moment in time;

F_0 – the fluorescence intensity at the initial time, when the pre-incubated ssDNA-gp32 is completely associated.

F_{max} - the fluorescence intensity of the gp32 protein alone, corresponding theoretically to the full dissociation of the ssDNA-gp32 complex.
